# Historical demography and genetic differentiation of the giant freshwater prawn *Macrobrachium rosenbergii* in Bangladesh based on mitochondrial and ddRAD sequence variation

**DOI:** 10.1002/ece3.3023

**Published:** 2017-05-09

**Authors:** M. M. Mahbub Alam, Kristen M. Westfall, Snæbjörn Pálsson

**Affiliations:** ^1^ Department of Life and Environmental SciencesUniversity of IcelandReykjavikIceland; ^2^ Faculty of FisheriesSylhet Agricultural UniversitySylhetBangladesh; ^3^ Pacific Biological StationFisheries and Oceans CanadaNanaimoBCCanada

**Keywords:** conservation, cytochrome oxidase subunit 1, management, population structure, single nucleotide polymorphisms

## Abstract

*Macrobrachium rosenbergii*, the giant freshwater prawn, is an important source of high quality protein and occurs naturally in rivers as well as commercial farms in South and South‐East Asia, including Bangladesh. This study investigated the genetic variation and population structure of *M. rosenbergii* sampled from four rivers in Bangladesh (sample size ranged from 19 to 20), assessing sequence variation, both in the mitochondrial cytochrome oxidase subunit 1 (CO1) gene and in 106 single nucleotide polymorphisms (SNPs) sampled randomly from the genome with double digest RAD sequencing (ddRADseq). The mitochondrial variation presented a shallow genealogy with high haplotype diversity (*h *=* *0.95), reflecting an expansion in population size for the last ~82 kyr. Based on the CO1 variation the current effective population size (*N*_e_) was 9.7 × 10^6^ (CI: 1.33 × 10^6^ – 35.84 × 10^6^) individuals. A significant population differentiation was observed with the mitochondrial CO1 sequence variation and based on the ddRADseq variation, which could be traced to the divergence of the population in the Naf River in the South‐East border with Myanmar from the other populations. A differentiation in mtDNA haplotype frequencies was also observed between the Biskhali River and the Karnaphuli Rivers in eastern Bangladesh. This study demonstrated the use of high‐throughput genotyping based on the ddRADseq method to reveal population structure at a small geographical scale for an important freshwater prawn. The information from this study can be utilized for management and conservation of this species in Bangladesh.

## 1 Introduction

*Macrobrachium rosenbergii* (De Man 1879; Decapoda, Palaemonidae), the giant freshwater prawn, is highly valued commercial aquaculture species. The species is found naturally in rivers and adjacent freshwater reservoirs (i.e. lakes, swamps, and canals) in South and South‐East Asia, from western Pakistan to western Java of Indonesia (FAO 2002; De Bruyn, Nugroho, Hossain, Wilson, & Mather, 2005; Hurwood et al., 2014). Because of its importance both for fishing and aquaculture, it has been introduced into 40 countries (Iketani et al., 2011). *Macrobrachium rosenbergii* has a catadromous life cycle. Copulation occurs in freshwater and ovigerous females migrate to estuaries, holding the fertilized eggs in the brood chamber, where eggs hatch as free‐swimming zoeae and after progressing through 12 larval stages in the brackish environment, the postlarvae (PL) enter into the freshwater system to grow until sexual maturity (FAO 2002, 2004).

During the last two decades, *M. rosenbergii* aquaculture has attracted considerable attention in Bangladesh for its export potential (Ahmed, Demaine, & Muir, 2008; Wahab, Ahmad‐Al‐Nahid, Ahmed, Haque, & Karim, 2012). Aquaculture operations have expanded in the South and South‐western districts of Bangladesh due to the availability of PL in the coastal areas (Azad, Lin, & Jensen, 2008). Moreover, a large number of freshwater ponds in Bangladesh have high potential to culture the freshwater prawn (Alam & Alam, 2014), which mostly depends on wild caught PL (Ahamed, Hossain, Fulanda, Ahmed, & Ohtomi, 2012; Ahmed, Occhipinti‐Ambrogi, & Muir, 2013; Ahmed & Troell, 2010). Concerns for the effects of wild prawn PL overfishing on coastal ecosystem biodiversity and production of other species caught as bycatch, led the Department of Fisheries (DOF) in Bangladesh to impose a ban in the year 2000 on wild prawn PL harvest (Ahmed & Troell, 2010; Department of Fisheries Bangladesh 2002). To fulfill the increased demand of PL, 27 freshwater prawn hatcheries have been established since 1992, producing about 27,000,000 PL in 2014 (Department of Fisheries Bangladesh 2015). Ovigerous females used in the hatcheries are collected directly either from the rivers or from aquaculture (Alam & Alam, 2014).

The genetic diversity of *M. rosenbergii* in Bangladesh is under continuous threat due to human activities (i.e. overexploitation, natural postlarvae collection, escape from aquaculture, and use of banned gears) and climate change effects (i.e. sea level rise, saline water intrusion) (Department of Fisheries Bangladesh 2013; Quader, 2010). Assessments of population structure and genetic diversity are essential to inform management of harvested populations as ignorance of structure can lead to overexploitation, and escapees from aquaculture can be a risk for locally adapted populations (Koljonen, 2001; Laikre, Palm, & Ryman, 2005; Olsson et al., 2007; Palsbøll, Berube, & Allendorf, 2007; Ward, 2000) as escaping from aquaculture occurred during cyclones and its resulted floods (Department of Fisheries Bangladesh 2008). Genetic diversity facilitates further evolution given environmental change and may in addition play a key ecosystem function (Reusch & Hughes, 2006). Information of genetic variation in *M. rosenbergii* in Bangladesh has until recently been mostly unexplored, but recent studies on Penaid shrimps in Bangladesh have uncovered high levels of genetic diversity (Alam, de Croos, & Pálsson, 2016; Alam & Pálsson, 2016; Alam, Westfall, & Pálsson, 2015, 2016; Hurwood et al., 2014). Several genetic studies have been applied to *M. rosenbergii* including allozymes (reviewed in Agarwal et al., 2016), mtDNA (De Bruyn et al., 2005; Hurwood et al., 2014), microsatellites (Hurwood et al., 2014; Khan et al., 2014), and more recently identification of single nucleotide polymorphisms (SNPs) (Agarwal et al., 2016; Jung et al., 2014). The analysis of mtDNA variation revealed three distinct lineages within the species, that is, a western lineage West of the biogeographic barrier at the Isthmus of Kra, a central lineage mainly from the Sunda‐Shelf region and an eastern lineage mainly in Indonesia (De Bruyn et al., 2005; Hurwood et al., 2014), but microsatellite variation showed four distinct clusters where Bangladesh samples clustered together with the samples from South‐western Thailand (Hurwood et al., 2014). A study on genetic patterns within *M. rosenbergii* sampled from South‐western Bangladesh (the Pashur and the Paira Rivers) and South‐eastern Bangladesh (the Naf River), based on seven microsatellites, did not reveal significant differences among the sites, but the pairwise distances corresponded with the geographical distances (Khan et al., 2014). The study by Agarwal et al. (2016) identified a high number of SNPs in transcribed regions of the *M. rosenbergii* genome sampled from India (0.16 to 6.02 per 100 bp), in comparison with other species. Another transcriptome study of this same species by Jung et al. (2014) found 2.5 SNP per 100 bp.

Double digest restriction‐site associated DNA sequencing (herein ddRADseq) and other variants of reduced‐representation sequencing have proven to be an effective tool to delineate genetic structure for various species, with a larger power than traditional genetic markers, by analyzing variation in thousands of loci, for example, in the American lobster *Homarus americanus* (Benestan et al., 2015) and three spine stickleback *Gasterosteus aculeatus* (Baird et al., 2008; Hohenlohe et al., 2010; Jones et al., 2012).

The aim of this study was to investigate the genetic variation and population structure of *M. rosenbergii* within Bangladesh by assessing genomic variation in samples from four of its main rivers: the Bishkhali in the West, the Meghna, and the Karnaphuli in the East and the Naf River at the boundary with Myanmar in South‐East by applying more genetic markers than in a previous study by Khan et al. (2014) and including other rivers. This was carried out by analyzing geographical and historical patterns in mitochondrial DNA sequences and from double digest restriction‐site associated DNA sequence (ddRADseq; Peterson, Weber, Kay, Fisher, & Hoekstra, 2012) variation.

## 2 Materials and Methods

### 2.1 Sample collection and DNA extraction

**Figure 1:**
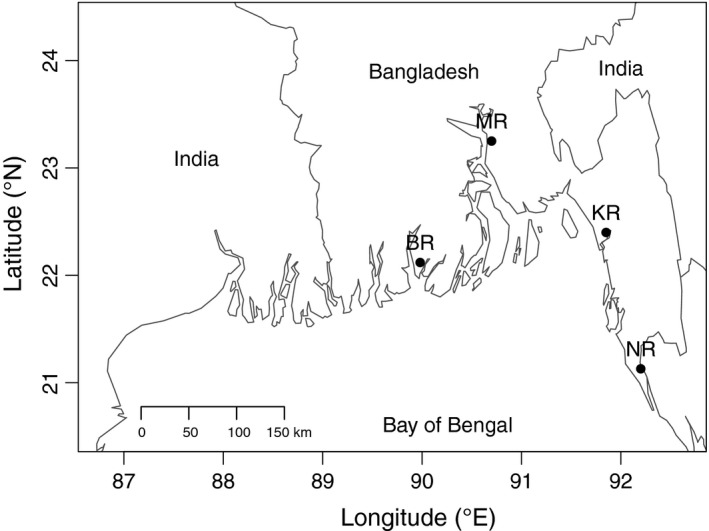
Sampling sites along the coast of Bangladesh. Capital letters indicate the four sampling sites–BR, Bishkhali River; MR, Meghna River; KR, Karnaphuli River; NR, Naf River

A total of 83 wild origin *M. rosenbergii* were collected during the period from December 2012 to September 2013 by artisanal fishermen from four rivers in Bangladesh; Bishkhali River (BR, 20 ind.), Meghna River (MR, 20 ind.), Karnaphuli River (KR, 22 ind.), and postlarvae (PL) from the Naf River (NR, 21 ind.) (Figure 1). All samples were preserved in 96% ethanol. Total genomic DNA was extracted for mtDNA sequencing from ~1 mg pleopod tissue through overnight incubation at 56°C in a mixture of 6% Chelex and 0.2 mg/ml proteinase K, using a Thermomixer (Eppendorf Thermomixer Compact), and for ddRADseq from ~20 mg pleopod tissue using standard Phenol–Chloroform extraction (Maniatis, Fritsch, & Sambrook, 1982). The quality and concentrations of DNA were examined with a ND‐1000 spectrophotometer using ND‐software (Thermo Fisher Scientific). For ddRAD sequencing 500 ng gDNA was allowed to run on 2% agarose gel to ensure quality. All gDNAs (1,000 ng) were treated with RNase to get rid of RNA before library preparation.

### 2.2 Mitogenomic variation

#### 2.2.1 Polymerase chain reaction (PCR) and sequencing

Variation in the mtDNA was assessed by sequencing a 1,316 bp fragment of the CO1 gene from 83 specimens (Genbank Accession numbers: KX585687–KX585769): corresponding with 132–1,447 bps of *M. rosenbergii* mitochondrial genome (GenBank accession number: AY659990.1; Miller, Murphy, Burridge, & Austin, 2005). The region includes the barcoding region (CO1b) and a downstream region (CO1d). The CO1b fragment was amplified using standard barcode primers LCO‐1490 and HCO‐2198 (Folmer, Black, Hoeh, Lutz, & Vrijenhoek, 1994), and the CO1d fragment with COIF (Palumbi & Benzie, 1991) and TL2N (Quan et al., 2001). PCR was performed in a volume of 10 μl, including 30–150 ng DNA, 0.2 mmol/L dNTP, 0.1% Tween 20, 1× Standard Taq Buffer (New England Biolabs), 0.5 mg Bovine Serum Albumin, 0.5 U Taq DNA Polymerase, and 0.34 mmol/L each of forward and reverse primers. The amplification protocol of CO1b fragment included an initial denaturation at 94°C for 4 min, and 37 cycles of denaturation at 94°C for 30 s, annealing at 45°C for 45 s, and extension at 72°C for 1 min, then a final extension at 72°C for 6 min. The PCR protocol for the CO1d region contained an initial denaturation at 94°C for 5 min, 38 cycles of denaturation at 94°C for 30 s, annealing at 55°C for 30 s, and extension at 72°C for 30 s, then a final extension at 72°C for 7 min.

All PCR products were examined on a 1.5% agarose gel with 1 μl Bromophenyl Blue and visualized under UV light, after staining with Ethidium Bromide. An ExoSAP reaction was performed to remove excess nucleotides and primers from PCR products (5 μl) in a 10 μl reaction volume. The DNA template (1 μl) was sequenced with the Big Dye Terminator kit 3.1 (AB), precipitated with ethanol and run on a Genetic Analyser (3500xL Applied Biosystems). The sequences were edited using BioEdit Sequence Alignment Editor (Hall, 1999) and aligned by applying the ClustalW Multiple alignment.

#### 2.2.2 Genetic diversity and population differentiation

Genetic diversity of the combined CO1 fragments of *M. rosenbergii*, including haplotype diversity (*h*), nucleotide diversity (π) and the partition among sample sites with analysis of molecular variance (AMOVA) applying both the distance method (Φ) and the conventional F‐statistics from haplotype frequencies were calculated using ARLEQUIN v3.5 (Excoffier & Lischer, 2011). Significance level of the genetic partition was tested by 1,000 permutations of individuals among samples. Haplotype richness (*H*_R_) was calculated using the allele richness function in HIERFSTAT package in R (Goudet, 2005). Evolutionary relationships were investigated with an unrooted cladogram, using a median‐joining algorithm (Bandelt, Forster, & Röhl, 1999), implemented in NETWORK v 5.0.0.0 (www.fluxus-engineering.com).

#### 2.2.3 Demographic history and population expansion

Population demographic changes and deviation from neutrality in *M. rosenbergii* were estimated by analyzing the mismatch distribution, using sum of square deviation (SSD) (Excoffier, 2004) and the raggedness index (Harpending, 1994), and with Tajima's D (Tajima,^1993^) and Fu's Fs (Fu, 1997) using ARLEQUIN v3.5 (Excoffier & Lischer, 2011). The time since expansion was based on the median of the mismatch distribution (τ) and the mutation rate, μ = 0.7 to 1.3% per site per Myr, for the CO1 (Knowlton & Weigt, 1998; Knowlton, Weigt, Solorzano, Mills, & Bermingham, 1993; Schubart, Diesel, & Hedges, 1998), as *t* = τ/(2 μl), where L is the length of the sequence. The demographic changes were further analyzed with the Bayesian Skyline Plot (BSP), based on the 83 CO1 sequences to estimate the past population dynamics from the time of sampling, assuming no population structure. The BSP analysis was implemented in BEAST v1.7.5 (Drummond, Ho, Rawlence, & Rambaut, 2007), following a strict molecular clock and the TN93 model with Invariants sites (I), derived from a PhyML Test (Guindon et al., 2010) using the APE package (Paradis, 2012) in R (R Core Team 2015). Posterior probability of the effective population size (*N*_e_) was estimated with the BSP analysis, using MCMC procedures by moving backward until the time of the most recent common ancestor was reached (Liao et al., 2010). Markov chains were run for 5.0 × 10^7^ generations and sampled every 1000. Log files were visualized for the posterior probabilities of the Markov Chain statistics using TRACER v1.5 (Rambaut & Drummond, 2009), and 10% of the samples were discarded as burn‐in during “skyline” reconstruction. Skyline data were exported from TRACER v1.5, and the skyline plot was redrawn using the package APE (Paradis, 2012) in R (R Core Team 2015).

### 2.3 Double digest RADseq variation

#### 2.3.1 Library preparation

Double digest RADseq library for 83 individuals was prepared following protocols modified from Peterson et al. (2012) and Elshire et al. (2011). Total gDNA (500 ng) was double digested using Sau3AI (1 U) and ApeKI (2 U), respectively in sequential incubations of 4 hr each, in NEB Buffer 4. The digested DNA (100 ng from each individual) was ligated to adapters containing a unique combination of two inline barcodes for each individual and complementary ends to the restriction overhangs (adapter sequence and barcodes from Elshire et al., 2011). Eleven unique barcodes on the Ape KI adapters (5 bps) and eight unique barcodes on the Sau 3AI adapters (6 bps), each pair with a minimum distance of two, were used to generate one unique combinatorial barcode for each individual. Adapters were used in molar excess, in approximately 6:1 molar ratio of each adapter to fragmented DNA, calculated from the median fragment size as assessed on agarose gel. Adapters were ligated using NEB T4 DNA ligase and supplied buffer at 21°C for 4 hr. The ligase was heat inactivated and then all individuals were pooled for size selection.

The pool of fragmented DNA, ligated with uniquely barcoded adapters, was purified applying magnetic beads (Macherey Nagel NGS Clean‐up and Size Select) following manufacturers protocol and eluted in sufficient volume of water for size selection. The fragmented DNA was size selected using a Pippin Prep (Sage Science) with 2% ethidium‐free gels and external size standard. Size selection used the narrow setting with a median fragment size of 400 bp (± 18 bp). The resulting fragments were amplified using PCR primers from Elshire et al. (2011) (final concentration 0.5 mmol/L) and NEB One Taq 2 × Master Mix with standard buffer. The Pippin Prep eluate was divided into eight separate PCR (10 μl per reaction). The PCR cycling conditions were 72°C, 3′; 98°C, 30″; (98°C, 10″; 65°C, 30″; 72°C, 30″) X 12; 72°C, 5′; 4°C, ∞. PCR products were purified using magnetic beads (Macherey Nagel NGS Clean‐up and Size Select) following manufacturers protocol. The PCR product was stained with SYBR Gold (Invitrogen) and quantified in Tecan (Genios) using a standard concentration curve generated from serial dilutions of lambda DNA (Table S1). Libraries were diluted 1:200 and 1: 400 and an average concentration calculated. Molarity was calculated based on the median fragment size of 400 bp. The library was run on an Illumina MiSeq2000 for 300 cycles (2 × 150 paired‐end) using v2 chemistry. Dilution and preparation for sequencing followed manufacturer's protocol, with the exception of a final library concentration of 38 p.m.

#### 2.3.2 Bioinformatics and genotyping

Raw FASTQ files from the MiSeq runs were demultiplexed into unique reads for each individual using *process_radtags* command in STACKS v.1.09 (Catchen, Hohenlohe, Bassham, Amores, & Cresko, 2013). Reads were truncated to 140 bp to obtain equal length of the sequences and filtered for overall quality of 90% (raw phred score 10). Individuals with <300,000 reads were discarded (*n* = 10). Variant detection and genotyping were performed using denovo pipelines: *ustacks*,* cstacks,* and *stacks* in STACKS v.1.09 (Catchen et al., 2013). Min depth of coverage to create a stack was 7, maximum distance allowed between stacks was 2 and to align secondary reads 4. Max number of stacks allowed per de novo locus was 3.

A table with single SNP per locus, selected among SNPs that had the least missing data per locus and read into R (R Core Team 2015) for statistical analysis. Deviation from random association of the variable sites within populations was calculated by comparing expected and observed heterozygosity, calculating the inbreeding coefficient (*F*_IS_) with bootstrapped confidence intervals and testing the deviation from Hardy–Weinberg, using the HIERFSTAT (Goudet, 2005) and PEGAS (Paradis, Jombart, Schliep, Potts, & Winter, 2016) packages in R. HIERFSTAT were also used to infer the population structure by calculating *F*_ST_ (Weir & Cockerham, 1984) across all population and for pairwise comparisons. The *F*_ST_ values were tested by 1000 permutation of individuals across samples. To evaluate the effect of loci which were not in Hardy–Weinberg equilibrium, whether due to natural selection or deviation from Mendelian segregations, the calculations were repeated by omitting those loci which failed the exact test within populations, applying the Fishers Combined probability (e.g. Sokal & Rohlf, 1995), with *p* < .05. To analyze further putative effect of selection, PGD Spider (Lischer & Excoffier, 2012) was used to convert the single SNP per locus file to BayeScan (Foll, 2012) format. Given enough statistical power BayeScan enables the identification of the effect of natural selection on the population subdivision at different loci, either due to diversifying or balancing/directional selection, which is characterized by positive and negative alphas, summarized using the package BOA (Smith, 2015) in R (R Core Team 2015).

Ordination of the SNP genotypes was investigated using discriminant analysis of principal components (DAPC), and followed by the assignment of individuals to different clusters defined with and without prior information, as implemented in the ADEGENET package (Jombart et al., 2015) in R.

## 3 Results

### 3.1 Mitochondrial DNA diversity

**Figure 2:**
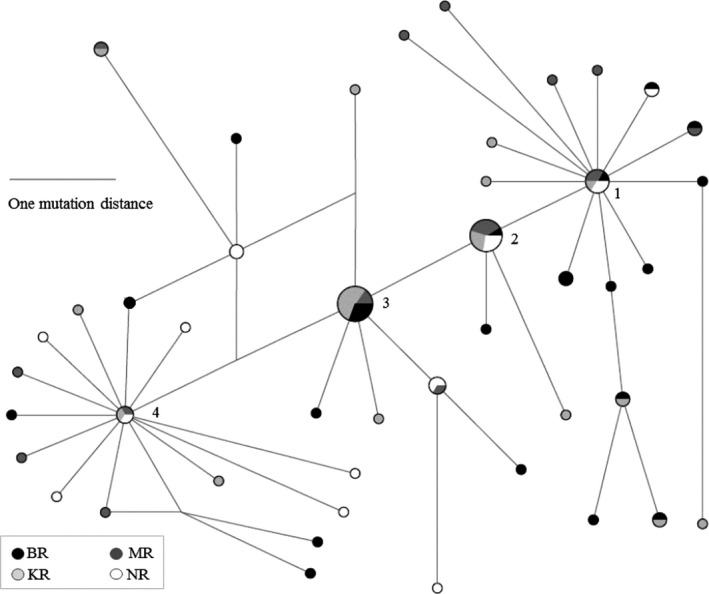
Median‐joining haplotype network based on mitochondrial CO1 (1,316 bps) of 83 *Macrobrachium rosenbergii* sampled from four rivers in Bangladesh. Each pie represents a haplotype and its size reflects the frequency. Distances between pies correspond to number of mutational differences between haplotypes. Shadings (black to white) denote four sampling locations, BR, MR, KR, and NR, respectively, see Figure 1)

The mitochondrial variation was characterized by a high overall haplotype diversity close to its maximum value (*h *=* *0.90–0.99), and nucleotide diversities ranged from 0.0022 to 0.0031 for the different samples (Table 1). The combined CO1 sequences produced 45 unique haplotypes from 83 individuals, of which 33 were singletons (Figure 2). Haplotypes 1 and 2 were found in all locations, representing 6 and 11 individuals respectively. Two haplotypes (Haplotypes 3 and 4), representing 13 and 3 individuals respectively, were found in three of the four sampling locations (Figure 2). One to two mutations distinguished most of the sequences from four common haplotypes, forming a shallow network that indicates recent ancestry within the mtDNA lineage (Figure 2).

**Table 1:** Genetic diversity in *Macrobrachium rosenbergii* from Bangladesh, based on mitochondrial CO1 gene (1,316 bps)

Sampling location		*N*	*N*_*h*_	*H*_R_	*h *± *SE*	π ± *SE*	S
BR	Bishkhali river	20	19	19	0.99 ± 0.02	0.0031 ± 0.0018	22
KR	Karnaphuli river	22	14	12.9	0.90 ± 0.06	0.0024 ± 0.0014	18
MR	Meghna river	20	15	15	0.96 ± 0.03	0.0024 ± 0.0015	18
NR	Naf river, Bangladesh‐Myanmar	21	13	12.6	0.94 ± 0.03	0.0022 ± 0.0014	15
All		83	45	14.8	0.95 ± 0.01	0.0026 ± 0.0015	46

*N,* No. of individuals; *N*_*h*_ *,* No. of haplotypes; *H*_R_, haplotype richness; *h,* haplotype diversity; π*,* nucleotide diversity; S, No. of segregating sites; *SE*, standard error.

Differentiation was observed among the samples along the coast of Bangladesh based on the CO1 sequences. Marginal difference was observed when genetic distances among haplotypes were considered (overall Φ_ST_ = 0.030, *p *=* *.045), but not for the haplotype frequencies (overall *F*_ST_ = 0.009, *p *>* *.05). The greatest differentiation occurred between the two most geographically distant locations, BR and NR (Φ_ST_ = 0.089, *p *=* *.012) and between BR and KR based on haplotype frequencies (*F*_ST_ = 0.042, *p *=* *.010) (Table 3).

**Figure 3:**
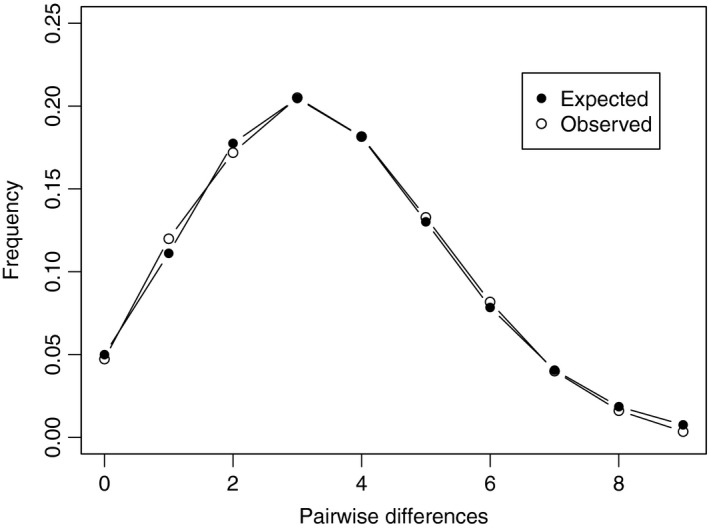
Mismatch distributions based on 83 sequences of mitochondrial CO1 (1,316 bps) of *Macrobrachium rosenbergii* from four rivers in Bangladesh, under the sudden expansion model

**Figure 4:**
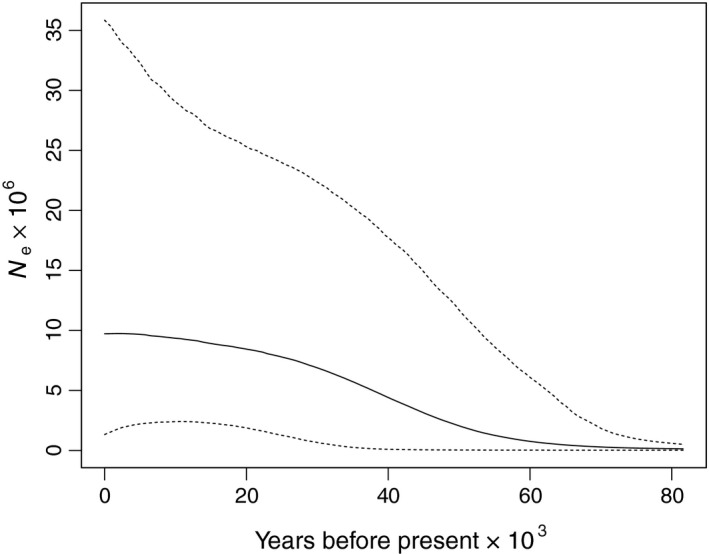
Bayesian Skyline Plot showing the past population dynamics of *Macrobrachium rosenbergii* in Bangladesh estimated from 83 sequences of mitochondrial CO1 (1**,**316** **bps). Dotted lines represent the 95% confidence intervals. Effective population size (*N*_e_ × 10^6^) per generation is traced back in time from the present to the past

The mismatch distribution for the samples (Figure 3) followed the sudden expansion model both for the SSD and the raggedness index (*p *>* *.98). Deviation from equilibrium was also observed with the Tajima's D and Fu's Fs, which were both negative and significant (Tajima's D = −2.035, *p *=* *.003; Fu's Fs = −26.220, *p *<* *.001), suggesting expansion in population size or range from a bottleneck or alternatively due to recovery from a selective sweep. The BSP analysis showed that the population has a current effective population size (*N*_e_) of 9.7 × 10^6^ (CI: 1.33 × 10^6^ – 35.84 × 10^6^) individuals and has undergone a steady increase in *N*_e_ for the last 81.6 kyr (with a lower confidence limit close to 35 kyr) (Figure 4). Time since expansion (*t*) of the Bangladesh *M. rosenbergii*, based on the median of the mismatch distributions (τ), was 100.35–186.37 kyr ago.

### 3.2 SNP Variation

#### 3.2.1 Genetic diversity

In total 141 stacks with 106 SNPs were obtained from the ddRADseq analysis of 73 individuals with an average depth of 18.6 (*SD* = 20.7) using the 7 depth base calling. The deviations of expected heterozygosity from observed heterozygosity (*F*_IS_) for the SNP dataset were overall negative, ranging from −0.22 to −0.66 within populations (Table 2). Nineteen loci were found to deviate from Hardy–Weinberg equilibrium, 17 with excess of heterozygotes (negative *F*_IS_ values from −0.55 to −1.00, (14 with *p* < .001) and two with positive *F*_IS_ values (0.88 and 1.00; *p *<* *.005). By omitting these 19 loci the overall heterozygosity decreased from 0.15 to 0.10, and the *F*_IS_ did not differ from 0 (Table 2). The expected heterozygosity for the single SNP dataset was similar among the sampling locations. (Table 2).

**Table 2:** Genetic diversity in *Macrobrachium rosenbergii* populations sampled from four rivers (BR, KR, MR, and NR; see Figure 1) in Bangladesh, based on 106 SNPs

Pop	*N*	*H*_e_	*F*_IS_(CI)	*H*_e_‐hw	*F*_IS__‐__hw_ (CI)
BR	17	0.14	−0.62/−0.33	0.12	−0.21/0.04
KR	20	0.14	−0.63/−0.37	0.13	−0.15/−0.01
MR	17	0.15	−0.54/−0.22	0.15	−0.09/0.10
NR	19	0.13	−0.66/−0.37	0.14	−0.22/−0.03
Total	73	0.14	−0.60/−0.34	0.08	−0.12/0.08

*N*, number of individuals; *H*_e_, expected heterozygosity; *F*_IS_, 95% confidence interval (CI) for the inbreeding coefficient obtained with bootstrap; *F*_IS‐hw_^,^ excluding loci which were not in Hardy–Weinberg equilibrium within samples.

#### 3.2.2 Detection of selection and demographic changes

Despite the deviation from Hardy–Weinberg equilibrium in some loci, variation at all SNP markers was in compliance with the neutral expectation obtained from BayeScan. The distribution of alpha values ranged from −0.06 to 0.51 with a mean of 0.00 and was all nonsignificant as the corrected *p* values (*q*) due to multiple testing using the FDR method (Foll, 2012) ranged from 0.66 to 0.90.

#### 3.2.3 Clustering of individuals and population differentiation

**Figure 5:**
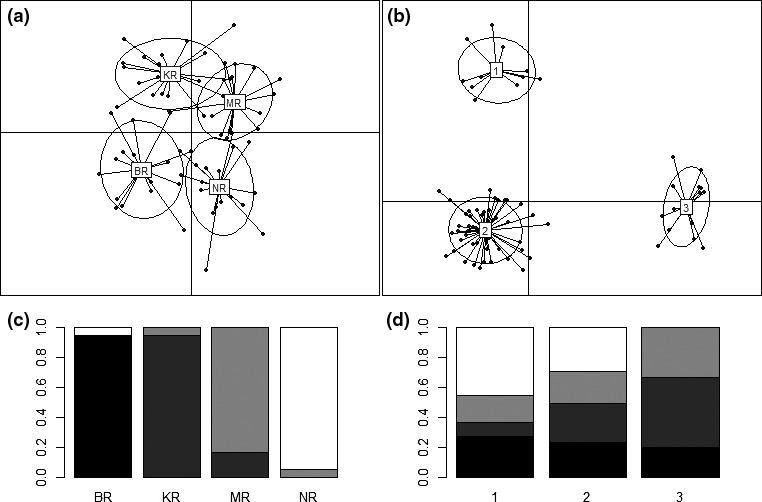
Discriminant analysis of principal components (DAPC) of *Macrobrachium rosenbergii*: (a) with prior information, (b) without prior information, (c) proportion of individuals in “a” from four locations (BR, KR, MR, and NR) and (d) proportion of individuals in “b” (clusters: 1, 2, and 3) sampled from four locations. Shadings (from black to white) represent four locations: BR, KR, MR, and NR, respectively

**Figure 6:**
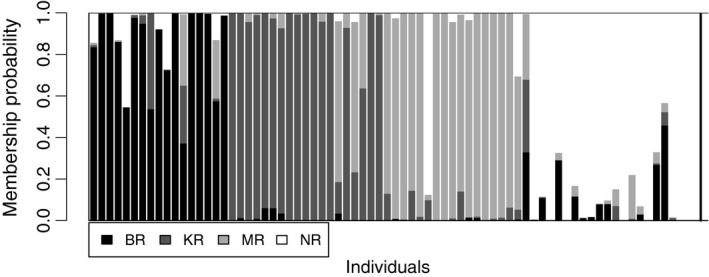
Assignment probabilities (*Q*‐values) of *Macrobrachium rosenbergii* individuals from the four sampling locations (BR, KR, MR, and NR; see Figure 1)

**Figure 7:**
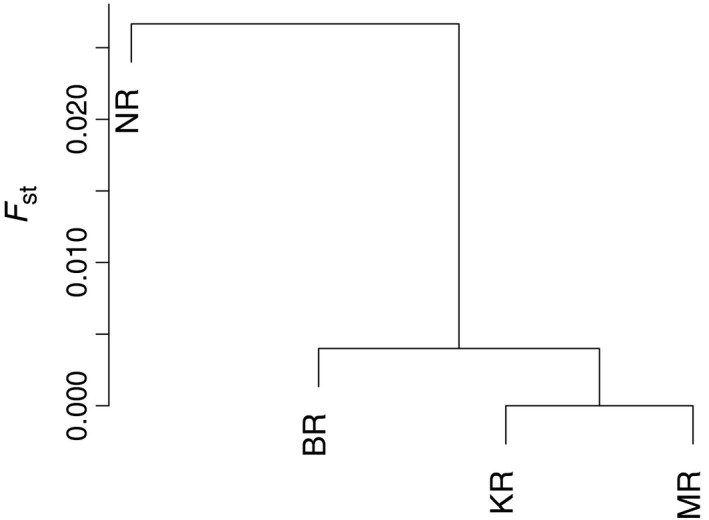
Differentiation among the four *Macrobrachium rosenbergii* populations along Bangladesh coastline. The dendrogram is based on the *F*_ST_ values, presented in Table 3, using the upgma method. Letters for sampling locations (BR, KR, MR, and NR) correspond to Figure 1

The DAPC analysis with prior information of the sampling sites, based on the SNP dataset, reveal distinct differentiation among populations, with the highest overlap between KR and MR (Figure 5a; see Figures 5c, and 6 for the proportion of individuals in each cluster). The DAPC analysis without the prior information resulted in three clusters (K = 3, stat = 86.25, alpha score = 0.16; Figure 5b). Individuals from BR, KR, and MR were found in all clusters but in different relative frequencies, but individuals from the NR in South‐eastern Bangladesh were only found in two of the clusters (Figure 5d). The population comparisons (*F*_ST_) for SNPs among all sampling locations were significant (*p* < .001, Table 3). The pairwise comparisons between populations among BR, KR, MR, and NR support the result observed from the DAPC analyzes that the NR differed most from the others (Figure 7). All comparisons with NR were significant (*p* < .02), whereas the others were not (Table 3). The pairwise differentiation between populations (*F*_ST_) was about two times larger when the loci which deviated from Hardy–Weinberg (similar values were observed when omitting all or just with excess of heterozygotes) were omitted, but the pattern remained the same. The differentiation between BR and KR, observed with the mtDNA, increased when omitting the loci which were not in HWE (*F*_ST_ = 0.013, *p* = .064).

**Table 3:** Pairwise *F*_ST_ and Φ_ST_ based on mtCO1 and 106 SNPs with corresponding *p* values between *Macrobrachium rosenbergii* populations sampled from four rivers (BR, KR, MR, and NR; see Figure 1) in Bangladesh. *p* values were obtained with 1,000 permutation of individuals across sites. *F*_ST‐_ _nhw_ and *P*_‐nhw_ are based on results where loci which were not in HWE within populations were excluded from the dataset

Pop	mtCO1				SNPs			
	*F*_ST_	*p*	Φ_ST_	*p*	*F*_ST_	*p*	*F*_ST‐_ _nhw_	*P*_‐nhw_
BR‐KR	0.042	.009	0.042	.058	0.003	.168	0.013	0.064
BR‐MR	0.006	.305	0.001	.340	0.005	.170	0.013	0.102
BR‐NR	0.017	.077	0.089	.012	0.011	.020	0.020	0.033
KR‐MR	0.005	.280	0.001	.367	0	.804	0	0.679
KR‐NR	0	.570	0.009	.258	0.034	<.001	0.076	<0.001
MR‐NR	0	.878	0.028	.111	0.035	<.001	0.072	<0.001
Overall	0.009	.136	0.030	.045	0.011	<.001	0.024	<0.001

## 4 Discussion

Information about genetic population structure and connectivity of natural populations is important for sustainable harvest of populations and the management of diversity (Olsson et al., 2007). Natural populations can be affected by human activities such as aquaculture, exploitation for consumption, and environmental changes. Application of next‐generation sequencing (e.g. RAD sequencing) has proven to be successful to detect population patterns, for example, in American lobster *Homarus americanus* (Benestan et al., 2015) and three spine stickleback *Gasterosteus aculeatus* (Baird et al., 2008; Hohenlohe et al., 2010; Jones et al., 2012), but application of such intensive methods has been limited in the developing nations, particularly in South and South‐East Asia where both biodiversity and threats to biodiversity are prominent (Willette et al., 2014). By using ddRADseq SNPs in addition to mitochondrial CO1 sequence variation, we revealed high genetic variation and two distinct populations of *M. rosenbergii* from different watersheds in Bangladesh: one in the Naf River, and the second in the Karnaphuli, the Meghna, and the Bishkhali rivers.

Analyzes of genome wide SNPs revealed a clear split between the population in the Naf River, at the boundary of Bangladesh and Myanmar, and the populations of the three other main rivers of Bangladesh which appear to be more connected, with an indication of gene flow and admixture among the rivers. Despite the divergence of the population in Naf River from other locations, two individuals (of 20) from the Naf River showed the closest genetic similarity with the individuals from the Bishkhali and the Meghna Rivers. This could possibly be explained by recent anthropogenic introductions, either due to transportation for aquaculture or accidentally through transfer of ballast water. The population sampled in Meghna River showed closest similarity with the Karnaphuli and the Bishkhali rivers, indicating connectivity and ongoing gene flow among these rivers either due to natural or human‐mediated mixing. Prawn can survive and grow in salinity up to 15 ppt (Chand et al., 2015), and possible migrations of larva, juveniles, and adults might have occurred among these rivers, through the Meghna and the Karnaphuli estuaries. Some extent of similarities was observed among all studied wild populations, which might have resulted from aquaculture practices as described earlier, or alternatively, due to shared ancestral polymorphism. Our findings were in line with the results from a previous genetic diversity study on *M. rosenbergii* sampled from the Pashur (36 ind.) and the Paira rivers (36 ind.), South‐West Bangladesh and the Naf River (36 ind.), South‐East Bangladesh, performed by Khan et al. (2014), based on seven variable microsatellites. Although their pairwise *F*_ST_ values (0.012–0.021) were not significant, they were almost similar to the values observed in our study (0.011–0.035). The detection of significant differentiation between the populations in the present study might have been supported by the larger number of the nuclear markers. The result from the DAPC ordination method supported the differentiation of the Naf River population but indicated that there might also be barriers to admixture between the rivers. When considering the combination of base pairs at the different sites within the genome, the most individuals sampled in one river were more similar to the individuals from the same river than to the individuals in other river.

To conclude, SNP variation revealed at least two distinct populations of *M. rosenbergii* sampled from Bangladesh: first, in the Naf River in between Bangladesh and Myanmar, and the second, in the Meghna, Bishkhali, and the Karnaphuli rivers. The haplotypic variation revealed high genetic variability within populations. Mitochondrial CO1 sequence variation revealed high overall haplotype diversity in Bangladesh *M. rosenbergii*, and revealed some extent of population differentiation, indicating similar pattern of population structure to the nuclear data, although the support was weaker. Based on our findings of these genetically distinct populations, we suggest that they should be considered as separate management units for sustainable management, harvest, and conservation. Fishing efforts can be controlled based on the genetic patterns to avoid over or underexploitation of different populations. As Bangladesh *M. rosenbergii* has high genetic variation, prawn hatcheries could be more sensible when they use ovigerous females from the same area in order to reduce threats to the local population diversity due to accidental escape from aquaculture. Further information about the biology of the species, such as variation in time of reproduction, behavior and habitat, is warranted to evaluate whether the observed population structure can be explained by different sources rather than the geographical origins.

## Conflict of Interest

None declared.
