# Supplementary figures and images for: Historical demography and genetic differentiation of the giant freshwater prawn Macrobrachium rosenbergii in Bangladesh based on mitochondrial and ddRAD sequence variation
Source: Ecol Evol. 2017 May 9;7(12):4326–35. doi: 10.1002/ece3.3023 (PMC5478082; doi:10.1002/ece3.3023)

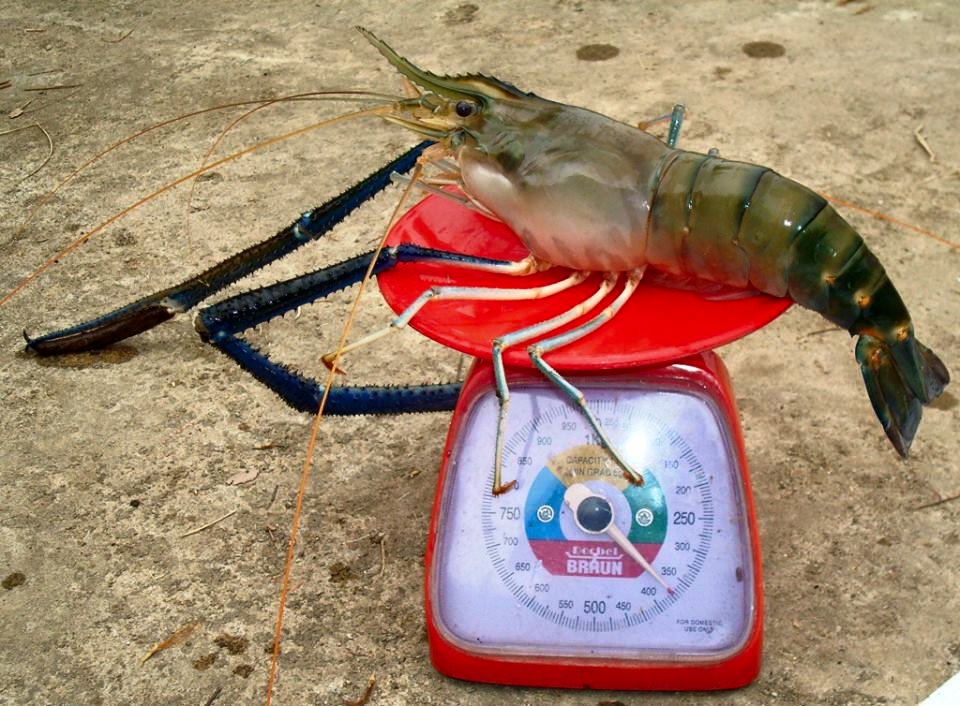

Supplement: Supplementary file 1 [file ECE3-7-4326-s001.jpg]
