# Supplementary material for: Historical demography and genetic differentiation of the giant freshwater prawn Macrobrachium rosenbergii in Bangladesh based on mitochondrial and ddRAD sequence variation
Source: Ecol Evol. 2017 May 9;7(12):4326–35. doi: 10.1002/ece3.3023 (PMC5478082; doi:10.1002/ece3.3023)
Supplement: Supplementary file 2 [file ECE3-7-4326-s002.docx]

Table S1. Serial dilution of lambda DNA in 1× TE. The left hand column is the concentration of the dilution (100 ul) and the right hand column is the final concentration in the assay after dilution with SYBR Gold (100 ul) to a total volume of 200 ul.

| dsDNA (ng/ml) | dsDNA (µl) | 1× TE | Total Volume (µl) | *in assay dsDNA (ng/ml) |
| --- | --- | --- | --- | --- |
| 2000.0 |  |  | 178.3 | 1000.0 |
| 1000.0 | 78.3 | 78.3 | 156.6 | 500.0 |
| 500.0 | 56.6 | 56.6 | 113.2 | 250.0 |
| 50.0 | 13.2 | 118.8 | 132.0 | 25.0 |
| 10.0 | 32.0 | 128.0 | 160.0 | 5.0 |
| 4.0 | 60.0 | 90.0 | 150.0 | 2.0 |
| 2.0 | 50.0 | 50.0 | 100.0 | 1.0 |
| *In assay describes the final concentration of dsDNA that is present. | | | | |
